# The Structural Basis of Oncogenic Mutations G12, G13 and Q61 in Small GTPase K-Ras4B

**DOI:** 10.1038/srep21949

**Published:** 2016-02-23

**Authors:** Shaoyong Lu, Hyunbum Jang, Ruth Nussinov, Jian Zhang

**Affiliations:** 1 Department of Pathophysiology, Key Laboratory of Cell Differentiation and Apoptosis of Chinese Ministry of Education, Shanghai Jiao Tong University, School of Medicine, Shanghai, 200025, China; 2 Cancer and Inflammation Program, Leidos Biomedical Research, Inc., Frederick National Laboratory, National Cancer Institute, Frederick, MD 21702, USA; 3 Department of Human Genetics and Molecular Medicine, Sackler School of Medicine, Sackler Institute of Molecular Medicine, Tel Aviv University, Tel Aviv 69978, Israel; 4 Institute of Bioinformatics and Medical Engineering, School of Electrical and Information Engineering, Jiangsu University of Technology, Changzhou 213001, China

## Abstract

Ras mediates cell proliferation, survival and differentiation. Mutations in K-Ras4B are predominant at residues G12, G13 and Q61. Even though all impair GAP-assisted GTP → GDP hydrolysis, the mutation frequencies of K-Ras4B in human cancers vary. Here we aim to figure out their mechanisms and differential oncogenicity. In total, we performed 6.4 μs molecular dynamics simulations on the wild-type K-Ras4B (K-Ras4B^WT^-GTP/GDP) catalytic domain, the K-Ras4B^WT^-GTP–GAP complex, and the mutants (K-Ras4B^G12C/G12D/G12V^-GTP/GDP, K-Ras4B^G13D^-GTP/GDP, K-Ras4B^Q61H^-GTP/GDP) and their complexes with GAP. In addition, we simulated ‘exchanged’ nucleotide states. These comprehensive simulations reveal that in solution K-Ras4B^WT^-GTP exists in two, active and inactive, conformations. Oncogenic mutations differentially elicit an inactive-to-active conformational transition in K-Ras4B-GTP; in K-Ras4B^G12C/G12D^-GDP they expose the bound nucleotide which facilitates the GDP-to-GTP exchange. These mechanisms may help elucidate the differential mutational statistics in K-Ras4B-driven cancers. Exchanged nucleotide simulations reveal that the conformational transition is more accessible in the GTP-to-GDP than in the GDP-to-GTP exchange. Importantly, GAP not only donates its R789 arginine finger, but stabilizes the catalytically-competent conformation and pre-organizes catalytic residue Q61; mutations disturb the R789/Q61 organization, impairing GAP-mediated GTP hydrolysis. Together, our simulations help provide a mechanistic explanation of key mutational events in one of the most oncogenic proteins in cancer.

Ras proteins are quintessential members of small GTPases that function as molecular switches by alternating between inactive GDP-bound and active GTP-bound states^1^,^2^. Activation is tightly regulated by guanine nucleotide exchange factors (GEFs), which catalyze the exchange of GDP by GTP^3^,^4^. Recent study uncovered a higher level of complexity of Ras activation at the membrane^5^. Active Ras-GTP can bind and activate downstream effectors, including Raf kinase, phosphatidylinositol 3-kinase (PI3K) and Ral guanine nucleotide dissociation stimulator (RalGDS), to promote cellular proliferation, survival, growth and differentiation^6^,^7^,^8^,^9^,^10^. Ras inactivation is mediated by GTPase-activating proteins (GAPs) which augment the intrinsic GTP hydrolysis rate of Ras by several orders of magnitude^11^,^12^,^13^,^14^. Ras mutations that impair GTPase activity are insensitive to GAPs rendering mutant Ras proteins persistent in their GTP-bound active state, thereby prolonging downstream signaling associated with oncogenic cell growth. Oncogenic mutations in Ras are found in approximately 30% of human cancers^15^.

The three human Ras genes encode four proteins: H-Ras, N-Ras and the splice variants K-Ras4A and K-Ras4B. All share approximately 90% sequence identity in their catalytic domain (residues 1–166) but show significant differences in their C-terminal hypervariable region (HVR)^16^. Post-translational modification of HVR is required for insertion of the HVR into the cellular membrane^17^. Despite a high degree of similarity across Ras isoforms, the frequency and distribution of Ras mutations are not equivalent. The Catalog of Somatic Mutations in Cancer (COSMIC) confirms that K-Ras is the most frequently mutated isoform in Ras-driven cancers (86%), followed by N-Ras (11%) and H-Ras (3%)^15^. Significantly, 98% of oncogenic Ras mutations are found at active site amino acid residues G12, G13 and Q61, whose mutations impair intrinsic and GAP-mediated GTP hydrolysis. Nevertheless, cancer-associated Ras isoforms exhibit an intimate link to residue substitutions^15^,^18^. K-Ras G12 mutations (89%) are predominant in human cancers, followed by G13 (9%) and Q61 (1%) mutations. Moreover, the G12D mutation is arguably the most prevalent mutation among three frequent G12C (14%), G12D (36%) and G12V (23%) mutations. In addition, G13D (7%) and Q61H (0.6%) mutations are also observed.

Despite intense interest in Ras over the years^19^,^20^,^21^,^22^, targeting oncogenic Ras mutants remains a formidable task and they are still ‘undruggable’^23^. Recently, based on real-time NMR spectroscopy, Smith *et al*.^24^ showed that different oncogenic mutations have distinct abilities to affect the intrinsic GTP hydrolysis and nucleotide exchange. Here, due to the five-fold difference in rates as compared to GAP-catalyzed hydrolysis we do not explore intrinsic hydrolysis. The prohibitive time scales also prevent us from following nucleotide exchange. Instead, our nucleotide exchange simulations start from the already exchanged states aiming to identify the conformational transitions of the switch I and switch II domains during the GDP/GTP exchange.

Over the years molecular dynamics (MD) simulations have been carried out to understand the structure and function of wild-type (WT) and mutated proteins^25^,^26^,^27^, particularly those cancer-related proteins^28^,^29^,^30^,^31^,^32^,^33^. These have shown that the landscape of the proteins changes following mutations^34^,^35^,^36^. For the Ras proteins, MD simulations illustrated differential dynamics of the H-, K- and N-Ras in their GDP- and GTP-bound states^37^,^38^,^39^. The targeted MD simulations identified the conformational transition pathway from the active GTP-bound and inactive GDP-bound states of WT H-Ras^40^,^41^. Recent studies focused on mechanisms by which certain mutations, e.g. G12V, G13V, and Q61H, affect the Ras’ intrinsic GTPase activity^42^,^43^,^44^,^45^. In addition, previous MD simulations of WT H-Ras-GTP/GAP complex on very short timescales (~1 ns) revealed that Q61 of H-Ras and R789 of GAP played a pivotal role in GTP hydrolysis^12^,^46^. However, the extremely short MD simulations were unable to capture the impact of GAP binding on the conformational transitions of Ras. Currently, relatively little is understood about the key determinant involved in the impairment of GAP-mediated GTP hydrolysis by oncogenic mutations, and the mutational biases of Ras in human cancers have been unresolved.

Here, we address these questions by performing μs explicit solvent MD simulations of K-Ras4B catalytic domain in its GTP/GDP-bound states as well as the GTP-bound K-Ras4B–GAP complex of the WT and oncogenic states (G12C, G12D, G12V, G13D and Q61H mutations). The simulations show that in solution, K-Ras4B^WT^–GTP exists in two states: active and inactive. Oncogenic mutations elicit an inactive-to-active conformational transition in K-Ras4B-GTP. During our K-Ras4B^WT^-GDP simulations we did not capture conformations visiting the active state. However, the G12C and G12D mutations dramatically affect the distribution of the K-Ras4B inactive state and increase the exposure of the nucleotide-binding site. The exchanged nucleotide simulations indicate that GTP-to–GDP exchange in K-Ras4B^WT^ can sample the GDP-like open conformation, whereas in our timescales the GDP-to-GTP exchange simulation is incapable of capturing the GTP-like closed conformation. Finally, simulations of the K-Ras4B^WT^-GTP–GAP complex reveal that in addition to providing the arginine finger R789, GAP plays a key role in stabilizing the active, catalytically competent conformation of K-Ras4B. However, the oncogenic mutations markedly disturb the alignment of catalytic residue Q61 of K-Ras4B and GAP’s R789, impairing the GAP-mediated GTP hydrolysis. Collectively, our data provide detailed mechanisms of how oncogenic mutations affect the structural and dynamic behavior of K-Ras4B and resolve its mutational biases in human cancers.

## Methods

### Simulated Systems

In the simulation of K-Ras4B–GTP in aqueous solution, the structures of K-Ras4B^Q61H^ (PDB ID: 3GFT) and K-Ras4B^G12D^ (PDB ID: 4DSN)^47^ were extracted from the Protein Data Bank (PDB). In 3GFT, residue H61 was mutated back to native Q61 to represent the K-Ras4B^WT^-GTP and in 4DSN, residue S1 was mutated back to native M1 and the C-terminal HVR (residues 167–180) were deleted to model the K-Ras4B catalytic domain. The G13D mutant as well as the G12C and G12V mutants were constructed based on the 3GFT and 4DSN, respectively, by replacing targeted residues with desirable residues. The simulation of Q61H mutant was directly used the PDB 3GFT.

In the simulation of K-Ras4B–GDP, the structures of K-Ras4B^WT^ (PDB ID: 4LPK)^22^ and K-Ras4B^G12D^ (PDB ID: 4EPR)^48^ were selected. In the 4EPR, residue S118 was mutated to native C118. The G13D and Q61H mutants as well as the G12C and G12V mutants were constructed based on the 4LPK and 4EPR, respectively, by replacing targeted residues with desirable residues.

In the nucleotide exchange simulations, the last 400 ns snapshot of GTP/GDP-bound K-Ras4B^WT^ was extracted from the respective MD trajectory. Based on this simulated structure, GTP (GDP) in the nucleotide-binding site was replaced by GDP (GTP) and subsequently MD simulations were conducted on the resulting systems.

In the simulations of the K-Ras4B–GAP complex, the structure of H-Ras^WT^ in complex with GDP and AlF_3_ was extracted (PDB ID: 1WQ1)^49^. The substitutions Q95H, D107E, A121P, A121S, E126D, S127T, R128K, Y141F, E153D and Q165K of H-Ras were performed and the GDP and AlF_3_ were substituted by GTP to model the K-Ras4B^WT^-GTP–GAP complex. The sulfide bond between C771 and C876 in the GAP was defined. The respective mutants were then constructed by replacing targeted residues with desirable residues.

In the respective complexes, GppNHp, a nonhydrolyzable GTP analogue, was modified to physiological GTP. The parameters for GTP and GDP were taken from AMBER parameter database (www.pharmacy.manchester.ac.uk/bryce/amber). Energy minimization of the initial model was performed following the introduction of mutations in K-Ras4B. Next, the proteins were solvated in a truncated octahedral box with TIP3P^50^ water molecules; the box size was set to ensure a distance of at least 10Å between the protein and the box boundaries. Systems were neutralized using counterions.

### MD Simulations

MD simulations were performed using the AMBER 11^51^ package with the AMBER ff03 force field^52^. To remove bad contacts in the solvated systems, all systems were subjected to 2000 steps of the steepest descent energy minimization, followed by 3000 steps of the conjugate gradient energy minimization with a positional restraint of 500 kcal mol^−1^ Å^−2^ imposing on the heavy atoms of proteins. Subsequently, the entire system was minimized without any restraints. After minimization, each system was heated gradually from 0 K to 300 K within 300 ps. This was followed by constant temperature equilibration at 300 K for 700 ps, with a positional restraint of 10 kcal mol^−1^ Å^−2^ in the complex in a canonical NVT ensemble.

A total of 6.4 μs MD simulations were performed with periodic boundary conditions using the NPT ensemble; each K-Ras4B–GTP/GDP in their wild-type and mutated states was simulated for 400 ns, as well as each nucleotide exchange simulation and K-Ras4B–GTP/GDP–GAP in their wild-type and mutated states for 200 ns (Table S1). Langevin dynamics was used to maintain the temperature at 300 K with a collision frequency of 1 ps^−1^, and a Langevin piston was assigned to maintain the pressure at 1 atm. An integration step of 2 fs was set for the MD simulations. The long-range electrostatic interactions were incorporated by using the particle mesh Ewald method^53^ with a cubic fourth-order B-spline interpolation and by setting the direct sum tolerance to 10^−5^. A cut-off equal to 10 Å was used for short-range electrostatics and van der Waals interactions. The SHAKE method^54^, with a tolerance of 10^−5^ Å, was applied to constrain all covalent bonds that involve hydrogen atoms.

### Cross-correlation Analysis

To identify protein domains with correlated residue motions the cross-correlation coefficient, *C(i,j)*, for the displacement of all Cα atoms pairs, *i* and *j*, was calculated (E.g. 1),


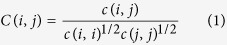


The value of *C(i,j)* is from −1 and 1. Positively correlated residues move in the same direction, whereas (negatively) anti-correlated residues in the opposite direction.

### Principal Component Analysis (PCA)

PCA^55^ was performed on a structural ensemble consisting of structures (snapshots every 20 ps) from the K-Ras^WT^-GTP. The covariance matrix *C* of the atomic coordinates was constructed (E.g. 2):


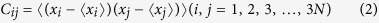


where *x*_*i*_ is a Cartesian coordinate of the *i*th C_α_ atom, 〈*x*_*i*_〉 represents the time average over all the configurations selected in the simulation, and *N* is the number of the C_α_ atoms. Prior to analysis, translation and rotational motions were excluded by overlaying the C_α_ atom of K-Ras^WT^-GTP to the reference crystal structure.

The diagonalization of *C* yields the eigenvalues *λ*_*i*_ and the corresponding eigenvectors *Vi*, namely, the principal component (PC). *Vi* represent the directions in the multidimensional space that correspond to independent modes of atomic motion, while *λ*_*i*_ represent their corresponding amplitudes. The first few PCs describe collective global motions in the protein. The projection Proj(*M*, PC_i_) of any structure (snapshot) *M* onto the *i*th PC was calculated (E.g. 3):


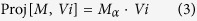


where *M*_α_ is the C_α_ atoms of proteins after overlaying *M* with the reference crystal structure.

### Cluster Analysis

The clustering was performed with the average-linkage algorithm that has been described previously^56^. The snapshots were superimposed using all C_a_ atoms to remove overall rotation and transition. Then, pairwise C_α_ atoms RMSD comparisons were performed between any snapshot and the average coordinate after rigid-body alignment using a threshold of 1.5 Å.

## Results

### Overview of K-Ras4B Structure and Simulations

**Figure 1:**
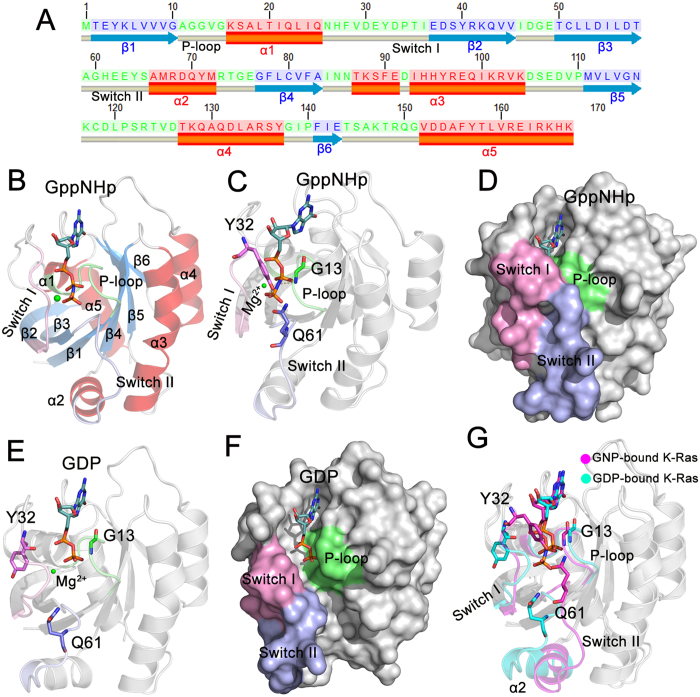
The architecture of GppNHp- and GDP-bound K-Ras4B catalytic domain. (**A**) The Kabsch-Sander secondary structure cartoon of the K-Ras4B catalytic domain. The blue solid arrows represent β-strands, the red solid cylinders represent α-helices and the gray solid cylinders represent loops. (**B**) Cartoon representation of crystal structure of GppNHp-bound K-Ras4B (PDB ID: 2PMX). The helices, strands and loops are colored by red, blue and gray, respectively. (**C**) Arrangements of active site residues G13, Y32 and Q61 in the GppNHp-bound K-Ras4B (**D**) Surface representation of GppNHp-bound K-Ras4B. (**E**) Arrangements of residues G13, Y32 and Q61 in the GDP-bound K-Ras4B (PDB ID: 4LPK). (**F**) Surface representation of GDP-bound K-Ras4B. (**G**) Backbone superimposition of GppNHp- (magenta) and GDP-bound (cyan) K-Ras4B. The P-loop, switch I and switch II domains, are colored by green, pink and light blue, respectively. Mg^2+^ ion is depicted by a green sphere.

K-Ras4B structure consists of two components, the catalytic domain (residues 1–166) and the membrane targeting HVR (residues 167–188)^57^. Previously, we revealed the effects of oncogenic mutations on the structural and dynamic characteristics of full-length K-Ras4B, particularly focusing on the HVR conformational behavior^58^,^59^. By contrast, the current study focuses on the catalytic domain. The catalytic domain is composed of six β-strands (β1–β6) flanked by five α-helices (α1–α5) and ten connecting loops (Fig. 1A,B). The functional P-loop (residues 10–17), switch I (residues 32–38) and switch II (residues 59–67) regions constitute the active site for GTP hydrolysis and interaction sites for effector proteins, including Raf, PI3K, RalGDS and GAP. Residues G13, Y32 and Q61 from the respective functional domain partake in H-bonding interactions with GTP (Fig. 1C), contributing to GTP hydrolysis. In the GTP-bound state, the three functional domains form the closed conformation of the GTP binding site (Fig. 1D). Following GTP → GDP hydrolysis, these domains relax into their open conformations (Fig. 1E,F), which allows GDP dissociation. Collectively, arguably the most remarkable differences in the GTP/GDP-bound K-Ras4B are in the switch I and switch II domains (Fig. 1G), revealing a crucial role of nucleotide-mediated cooperativity between the two switch lobes in the conformational transition. In particular, in the GTP-bound state, switch I residue Y32 is in the ‘up’ conformation that points to the γ-phosphate of GTP for hydrolysis, whereas in the GDP-bound state it undergoes a large flip and shifts to the interaction site where it resides in the ‘down’ conformation. Furthermore, the outward displacement of switch II after GTP hydrolysis results in the catalytic residue Q61 pointing away from the active site.

For the twenty systems of K-Ras4B in the different states we performed a total of 6.4 μs MD simulations (Table S1). Although the total MD timescales are significantly long, for each system the runs extend over 200 or 400 ns. If the individual simulation times were to extend to microseconds or milliseconds, we may observe the entire conformational transition pathway from the active GTP-bound to inactive GDP-bound states. These timescales are beyond the scope of current study. Indeed, remarkably our current simulation timescale can observe the conformational changes of K-Ras4B in its GTP/GDP-bound states induced by oncogenic mutations, which can explain how the oncogenic mutations affect the structural and dynamic behavior of K-Ras4B and impair the GAP-mediated GTP hydrolysis. First, each 400 ns simulation of K-Ras4B^WT^-GTP and its oncogenic mutants was carried out to explore the effect of oncogenic mutations. Second, each 400 ns simulation of K-Ras4B^WT^-GDP and the oncogenic mutants was deployed to probe how oncogenic mutations affect the dynamics of the nucleotide-binding site. Third, each 200 ns nucleotide exchange simulation of K-Ras4B^WT^ was conducted to investigate the conformational transitions and the accessibility of the two states during the nucleotide exchange process. Finally, each 200 ns simulation of K-Ras4B^WT^-GTP–GAP and the oncogenic mutants was performed to elucidate the role of GAP in catalysis and the disruption of GAP-mediated GTP hydrolysis in oncogenic mutants.

### Wild-type GTP-bound K-Ras4B Exists in Two States: Active and Inactive

**Figure 2:**
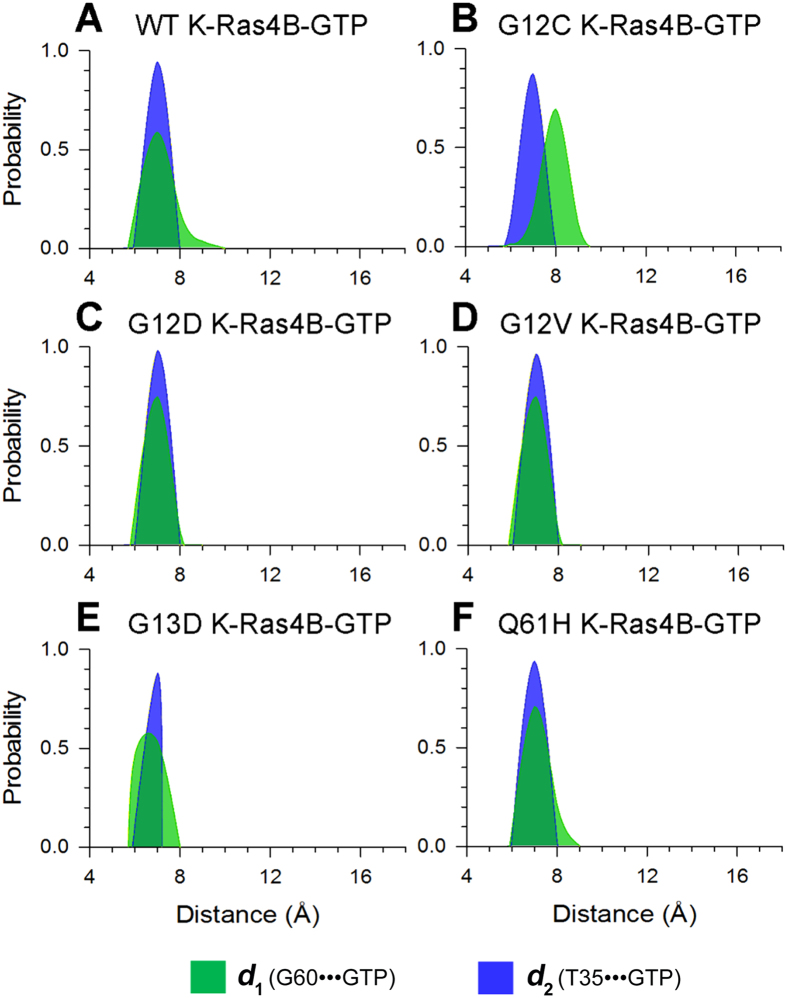
Oncogenic mutations shift the population of K-Ras4B–GTP from the inactive to the active state. The probability distributions for two atom-pairs distances, *d*_*1*_ (defined by the distance from G60 C_α_ atom to GTP P_β_ atom) and *d*_*2*_ (defined by the distance from T35 C_α_ atom to GTP P_β_ atom), were calculated on the MD snapshots of K-Ras-GTP. (**A**) wild-type, (**B**) G12C, (**C**) G12D, (**D**) G12V, (**E**) G13D and (**F**) Q61H mutants.

Recent ^31^P NMR spectra and crystal structures of guanosine 5′-(β,γ-imido)triphosphate (GppNHp)-bound forms of H-Ras mutants suggested that in their GTP-bound forms they exist in two interconverting conformations, ‘inactive’ and ‘active’^60^,^61^. The ‘active’ state is characterized by the stabilization of the switch I and switch II by the GTP through interaction of T35 and G60 with the γ-phosphate. The ‘inactive’ state contains two substates, 1 and 2; the former is described by both the disassociation of T35 and G60 from the γ-phosphate and the latter by only the loss of interaction of T35 with the γ-phosphate. The high sequence similarity between H- and K-Ras (sequence identity ~94%) raises the question of whether the two conformational states exist also in K-Ras4B^WT^-GTP. Principal component analysis (PCA) and cluster analysis show the existence of two, active and inactive, states in K-Ras4B^WT^-GTP (Fig. S1). However, the population of the active state is significantly larger than that of the inactive state in the GTP-bound form, in accordance with the two states distribution of H-Ras-GppNHp detected by NMR spectroscopy^61^. To delineate Ras conformations in the states, the probability distributions for two atom-pairs distances, one is defined by the distance from the C_α_ atom of switch II residue G60 to the P_β_ atom of GTP (*d*_1_) and the other from the C_α_ atom of switch I residue T35 to the P_β_ atom of GTP (*d*_2_), were calculated. As shown in Fig. 2, the probability distributions of *d*_2_ exhibit small differences between WT and mutated GTP-bound K-Ras4B, suggesting subtle conformational changes in the switch I domain triggered by oncogenic mutations compared to K-Ras4B^WT^-GTP. However, comparisons of the probability distributions of *d*_1_ between WT and mutated GTP-bound K-Ras4B reveal notable changes, indicating that oncogenic mutations have an effect on the conformational dynamics of switch II domain. For K-Ras4B^WT^-GTP, we observed two intervals of probability distributions of *d*_1_ (Fig. 2A); one with the *d*_1_ value from 5.8 Å to 8.0 Å and the other from 8.0 Å to 10.0 Å. An analysis of the representative structures of K-Ras4B^WT^-GTP corresponding to the two intervals indicated that both of T35-γ-phosphate and G60-γ-phosphate interactions existed in the former, which represents the active state (Fig. S2A), whereas a dearth of G60-γ-phosphate interaction was observed in the latter (Fig. S2B), which is significantly different from the two inactive substates observed in the GppNHp-bound H-Ras^60^. The newly identified conformer of K-Ras4B^WT^-GTP from our MD simulation may be designated as inactive substate 3. Collectively, these data suggest the existence of the active and inactive conformations of K-Ras4B^WT^-GTP in solution, in agreement with the conformational ensemble of GppNHp-bound H-Ras^60^. The three functional domains, P-loop, switch I and switch II, form the closed conformation of the nucleotide-binding site in the active state (Fig. S2A). Conversely, they are in the open conformation in the inactive substate 3 (Fig. S2B). Further backbone superimposition of the active state and the inactive substate 3 on the crystal structure of GppNHp-bound K-Ras4B indicates that the switch II domain undergoes a large conformational rearrangement in the inactive substate 3 compared to the active state and the crystal structure (Fig. S2C).

### Oncogenic Mutations Shift the GTP-bound K-Ras4B Ensemble to the Active State

Given that the conformational ensemble of K-Ras4B^WT^-GTP contains two active and inactive conformers, we next investigated the impact of oncogenic mutations on the conformational ensemble of K-Ras4B–GTP. For each oncogenic mutant, the probability distributions of *d*_1_ and *d*_2_ were calculated for the distances of the same atom-pairs. Compared to the plot of K-Ras4B^WT^-GTP (Fig. 2A), the probability distributions of *d*_1_, conspicuous in the G12D (Fig. 2C), G12V (Fig. 2D), and G13D (Fig. 2E) mutants, and to a lesser extent, in the Q61H mutant (Fig. 2F), are confined to the first interval (from 5.8 Å to 8.0 Å) of probability distributions of *d*_1_ in K-Ras4B^WT^. These data imply that conformers of oncogenic mutants predominantly exist in an active state, especially the G12D, G12V, and G13D mutants, and to a lower extent, the Q61H mutant. The conformational ensemble of the G12C mutant (Fig. 2B) still exists in two, active and inactive, states compared to the WT. These observations suggest that oncogenic mutations, except G12C, cause an inactive-to-active conformational transition in K-Ras4B^Mut^-GTP.

### Wild-type GDP-bound K-Ras4B Largely Exists in the Inactive State and G12C and G12D Mutants Display Larger Exposure of the Nucleotide-binding Site

**Figure 3:**
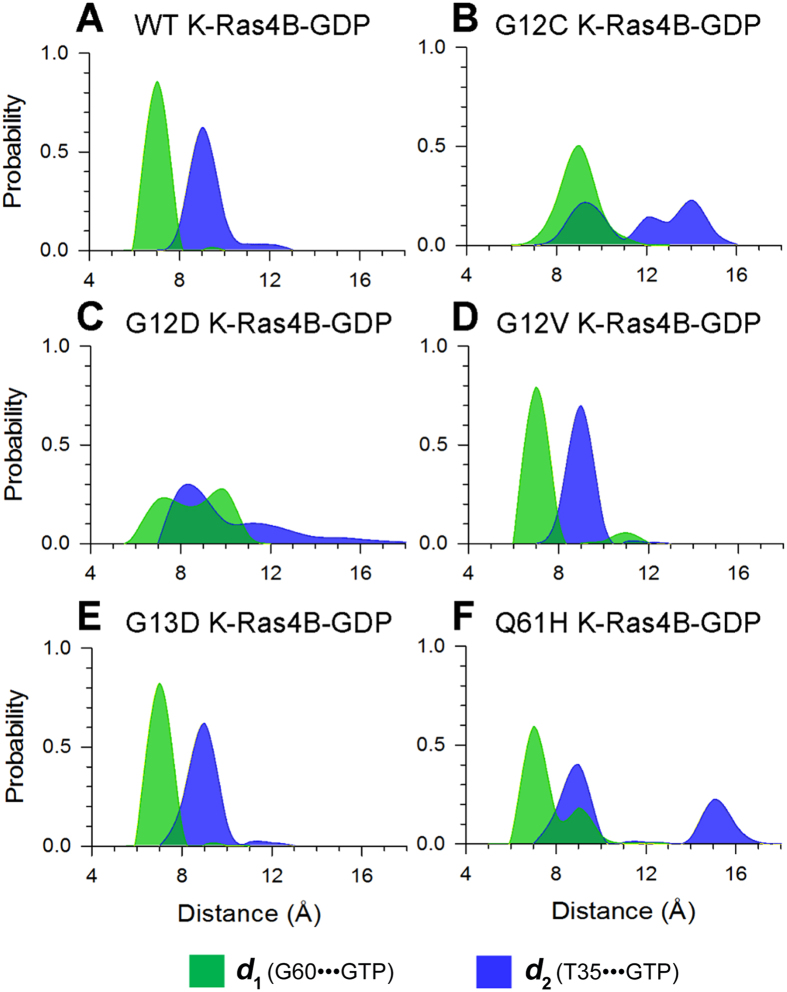
G12C and G12D mutations significantly affect the conformational ensemble of K-Ras4B-GDP. The probability distributions for two atom-pairs distances, *d*_*1*_ (defined by the distance from G60 C_α_ atom to GDP P_β_ atom) and *d*_*2*_ (defined by the distance from T35 C_α_ atom to GDP P_β_ atom), were calculated on the MD snapshots of K-Ras4B-GDP. (**A**) wild-type, (**B**) G12C, (**C**) G12D, (**D**) G12V, (**E**) G13D and (**F**) Q61H mutants. K-Ras4B^WT^-GDP exhibits one major energy-minima basin, corresponding to the inactive state. Oncogenic G12 mutations, particularly G12C and G12D, result in larger conformational changes of K-Ras4B-GDP.

**Figure 4:**
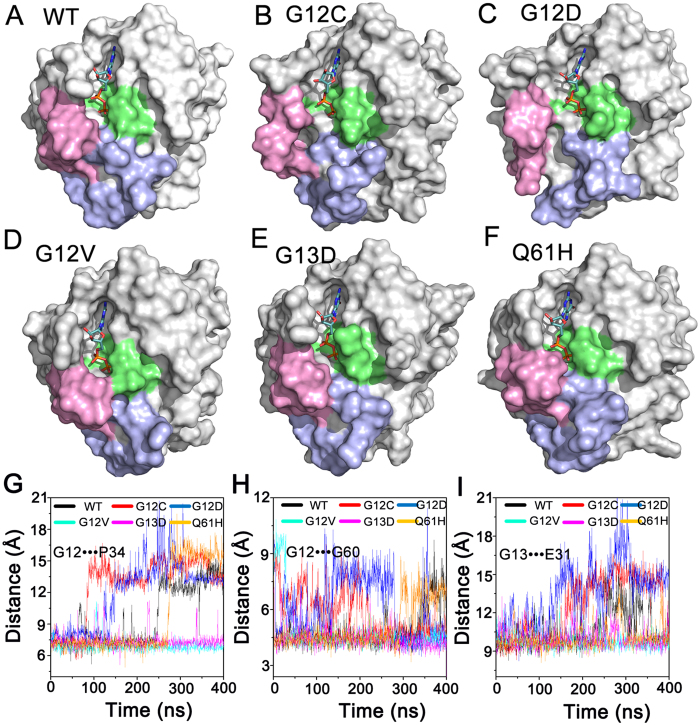
Oncogenic mutations affect the dynamics of GDP-binding site. Surface representation of the representative structures of the wild-type (**A**), G12C (**B**), G12D (**C**), G12V (**D**), G13D (**E**) and Q61H (**F**) mutants. Time dependence of interresidue distances between the C_α_ atoms of G12/P34 (**G**), G12/G60 (**H**), and G13/E61 (**I**) residue pairs. The oncogenic G12C and G12D mutations cause larger exposure of the nucleotide-binding site compared to the wild-type and other mutations.

**Figure 5:**
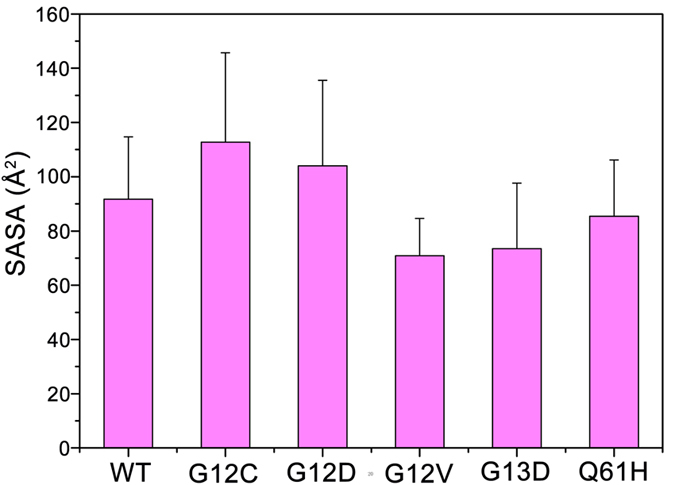
G12C and G12D mutations increase the exposure of the GDP-binding site. The solvent accessible surface area (SASA, Å^2^) of GDP in the wild-type and oncogenic mutants.

To reveal the conformational preference of K-Ras4B^WT^-GDP, the probability distributions for the atom-pairs distances, *d*_1_ and *d*_2_, were calculated (Fig. 3A). Comparison to the plot in the K-Ras4B^WT^–GTP (Fig. 2A), the probability distributions of *d*_1_ and *d*_2_, especially the *d*_2_, showed significant changes. Further analysis of a representative structure of K-Ras4B^WT^-GDP using cluster analysis indicated that the conformation represents the inactive state (Fig. 4A). Similarly, the probability distributions for the atom-pairs distances, *d*_1_ and *d*_2_, were calculated for the oncogenic mutants (Fig. 3B–F). Most remarkably, oncogenic G12 mutations, especially the G12C and G12D mutations and to a less extent, the Q61H mutant, result in larger conformational changes of K-Ras4B-GDP. Analysis of representative structures of each oncogenic mutant (Fig. 4B–F) revealed that the nucleotide-binding site is in more open conformation in the G12C and G12D mutants than in the K-Ras4B^WT^-GDP (Fig. 4A). To quantitatively monitor the dynamics of the nucleotide-binding site induced by oncogenic mutations, we measured the interresidue distances. Three pairs of distances were used: the combination of G12/P34 (Fig. 4G) and G12/G60 pairs (Fig. 4H) measures the motion of the phosphate-binding site of GDP, and the G13/E31 pair (Fig. 4I) measures the motion of the ribose-binding site of GDP. The analysis showed that the three interresidue distance pairs markedly augment in the G12C and G12D mutants compared to other oncogenic mutants and K-Ras4B^WT^, in agreement with the representative structural analysis. Furthermore, the solvent accessible surface area (SASA) of GDP was measured to predict the magnitude of mutations-induced conformational changes of the nucleotide-binding site in K-Ras4B^WT^ and its oncogenic mutants. As shown in Fig. 5, compared to the WT, the SASA of GDP in the G12C and G12D mutants increases by approximately 23% and 14%, respectively. Collectively, these data suggest that the oncogenic G12C and G12D mutations cause larger exposure of the nucleotide-binding site.

### Nucleotide Exchange Simulations

**Figure 6:**
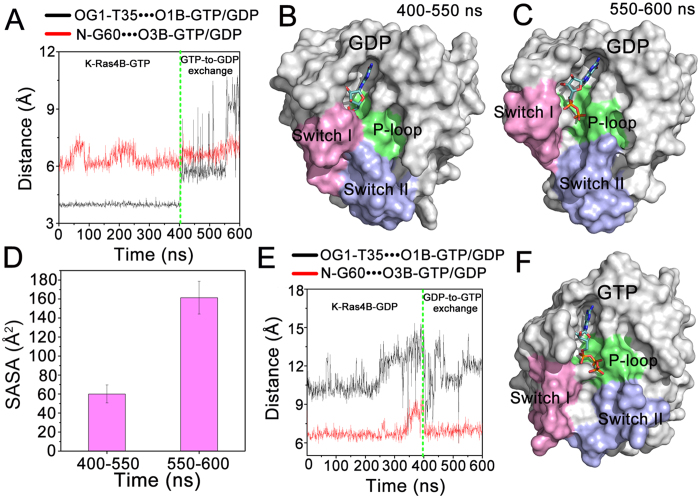
The conformational transition of K-Ras4B is more accessible in the GTP-to-GDP exchange than in the GDP-to-GTP exchange. (**A**) Time dependence of distances between T35 OG1 atom and GDP O1B atom as well as between G60 N atom and GDP O3B atom in both the K-Ras4B^WT^-GTP simulation (0–400 ns) and the GTP-to-GDP exchange simulation (400–600 ns). Surface representation of the representative structures of K-Ras4B derived from 400–550 ns (**B**) and 550–600 ns (**C**). (**D**) The SASA (Å^2^) of GDP in the periods of 400–550 ns and 550–600 ns. (**E**) Time dependence of distances between T35 OG1 atom and GTP O2G atom as well as between G60 N atom and GTP O1G atom in both the K-Ras4B^WT^-GDP simulation (0–400 ns) and the GDP-to-GTP exchange simulation (400–600 ns). (**F**) Surface representation of the representative structure in the period of 400–600 ns.

The structures of GTP/GDP-bound K-Ras4B emphasize the significant conformational differences of switch I and switch II regions (Fig. 1G). In the K-Ras4B–GTP, switch I and switch II are fixed, interacting with the γ-phosphate via T35 and G60 (Fig. S3A), respectively. In striking contrast, loss of these interactions is observed in the K-Ras4B-GDP (Fig. S3B), leading to the marked deviation of switch I and switch II from the active site. To identify the conformational transitions of the switch I and switch II during the GDP/GTP exchange, nucleotide exchange simulations were performed on K-Ras4B^WT^ using the last 400 ns snapshots of K-Ras^WT^-GTP/GDP by the replacement of GTP (GDP) in the nucleotide-binding site with GDP (GTP), respectively (see Materials and Methods). As shown in Fig. 6A, by monitoring the distances between T35, G60 and β-phosphate of GDP, the simulation of GTP-to-GDP exchange (400–600 ns) revealed that the distance between T35 and β-phosphate of GDP increases to 9.27 ± 0.94Å during the 550–600 ns compared to that of 5.88 ± 0.80Å in the period of 400–550 ns, while the distance between G60 and β-phosphate of GDP remains stable throughout the simulation. The representative structures extracted from the MD trajectory using cluster analysis exhibited that the nucleotide-binding site is in the closed conformation during the 400–550 ns (Fig. 6B), and it relaxes to the open conformation during the 550–600 ns (Fig. 6C). Consistently, the analysis of SASA of GDP showed that it markedly increases to 161.4 ± 17.2Å^2^ during the 550–600 ns compared to that of 60.4 ± 9.1Å^2^ during the 400–550 ns (Fig. 6D). Backbone superimposition of the two representative structures on the crystal structures of GppNHp/GDP-bound K-Ras4B indicated the outwardly displaced conformation of switch I in the representative structure derived from the 550–600 ns (Fig. S3C). The movement of the switch I during the 550–600 ns increases the conformational space between the switch I and GDP, which favors the flip of Y32 from the ‘up’ to the ‘down’ conformations during the nucleotide exchange. Therefore, this structure may represent an intermediate during the GTP-to-GDP transition. However, the simulation of GDP-to-GTP exchange suggested that despite the exchange of GDP with GTP, the conformers of K-Ras4B are still in the GDP-like open conformation throughout the whole simulation (Fig. 6E,F and S3D). Taken together, these data indicate that K-Ras4B with the GTP-to-GDP exchanged can sample the GDP-like open conformation, while in the GDP-to-GTP exchange sampling the GTP-like closed conformation is rare, which further implies that the conformational transition of K-Ras becomes more accessible in the GTP-to-GDP exchange than in the GDP-to-GTP exchange.

### GAP Stabilizes the Active, Catalytically Competent Conformation of Wild-type GTP-bound K-Ras4B

**Figure 7:**
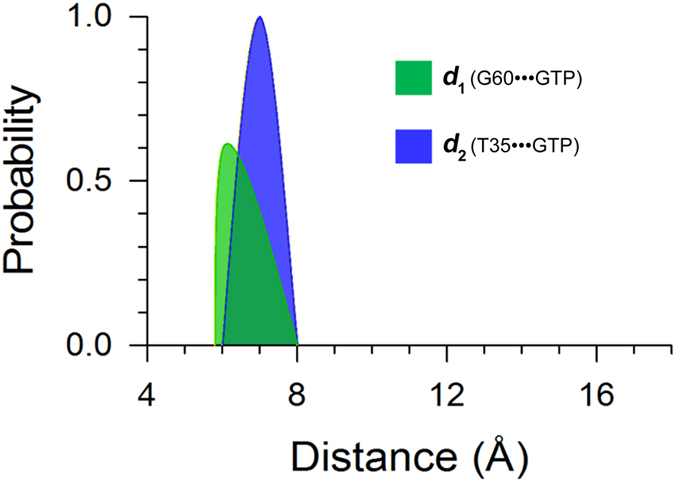
GAP binding shifts the conformational ensemble of K-Ras4B^WT^-GTP to the GTP-bound active state. The probability distributions for two atom-pairs distances, *d*_*1*_ and *d*_*2*_, were calculated on the MD snapshots of K-Ras4B^WT^-GTP–GAP.

**Figure 8:**
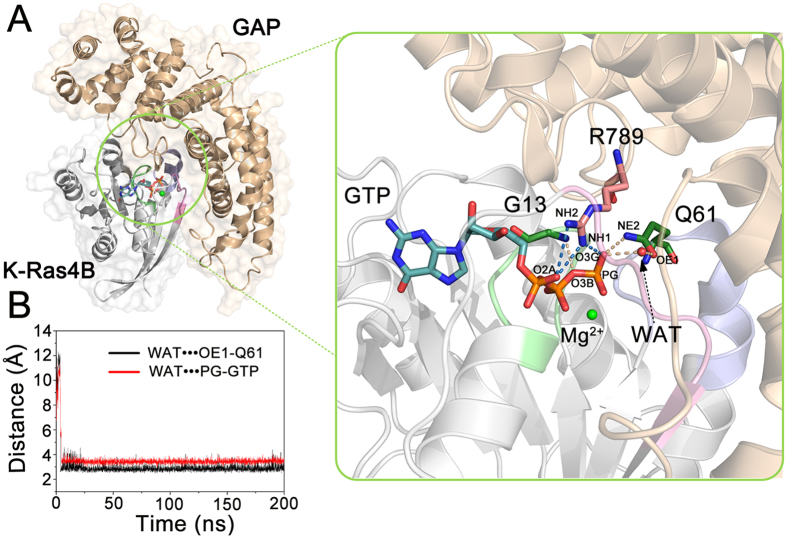
GAP stabilizes the active, catalytically competent conformation of K-Ras4B^WT^–GTP. (**A**) Details of interactions of K-Ras4B with GAP highlighting catalytically important elements. In the K-Ras4B^WT^–GTP complex, GAP provides the arginine finger R789 to interact with the α- and γ-phosphates of GTP. Meanwhile, the side chain carbonyl group of catalytic residue Q61 interacts with the catalytic water. In this conformer, Q61 can extract a hydrogen atom from the catalytic water, and the developing negative hydroxyl ion can attack the γ-phosphorus of GTP resulting in GAP-mediated GTP hydrolysis. (**B**) The evolution of the catalytic water molecule (WAT) to Q61 and GTP in the active site.

K-Ras4B^WT^-GTP exists in the active and inactive states. To unearth the effect of GAP on the conformational dynamics of K-Ras4B^WT^-GTP, the probability distributions for the atom-pairs distances, *d*_1_ and *d*_2_, were calculated. When binding to GAP, K-Ras4B^WT^-GTP exhibits one interval of probability distributions of *d*_1_ and *d*_2_ in solution (Fig. 7), with the *d*_1_ and *d*_2_ values from 5.8 Å to 8.0 Å and from 6.0 Å to 8.0 Å, respectively, which represents the GTP-bound active state. In the active site of K-Ras4B (Fig. 8A), G13 partakes in a H-bond with the β-γ bridging oxygen atom of GTP, Q61 that interacts with the catalytic water forms a H-bond with the γ-phosphate of GTP, and GAP provides the arginine finger R789 that protrudes into the active site of K-Ras4B to neutralize developing negative charges in the transition state via the salt bridge interactions with the α- and γ-phosphates of GTP. In this structure, the side chain carbonyl group of catalytic residue Q61 extracts a hydrogen atom from the catalytic water, and subsequently the negative hydroxyl ion can attack the γ-phosphorus of GTP to perform GAP-mediated GTP hydrolysis. Analysis of the MD trajectory revealed that the catalytic water diffuses into the active site after several ns simulation and then it forms stable water-mediated H-bonding interactions with the Q61 and γ-phosphate of GTP (Fig. 8B).

**Figure 9:**
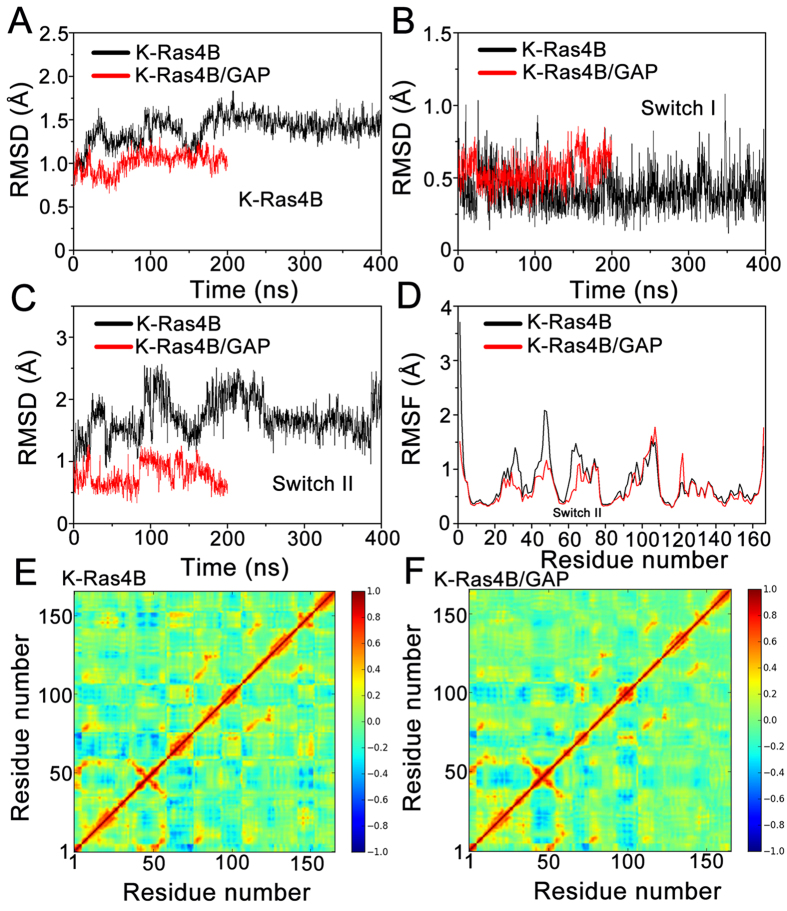
GAP stabilizes the catalytically-competent conformation of K-Ras4B in addition to providing the arginine finger R789 for catalysis. (**A**) The Cα atoms RMSD of K-Ras4B in the free K-Ras4B and K-Ras4B–GAP complex. The Cα atoms RMSD of K-Ras4B switch I (**B**) and switch II (**C**) regions in the free K-Ras4B and K-Ras4B–GAP complex. (**D**) The Cα atoms RMSF of K-Ras4B in the free K-Ras4B and K-Ras4B–GAP complex. The extent of correlation for all residue pairs (of Cα atom displacement) of K-Ras4B in the free K-Ras4B (**E**) and K-Ras4B–GAP complex (**F**). The domain-domain motions are markedly restricted in the K-Ras4B–GAP complex compared to the free K-Ras4B, particularly in the switch II domain (residues 59–67).

To further validate the stabilization of K-Ras4B by GAP binding, the root-mean square deviations (RMSD) of K-Ras4B in the free and complexed with GAP states were analyzed. As shown in Fig. 9A, GAP markedly stabilizes the global dynamics of K-Ras4B in the K-Ras4B–GAP complex compared to free K-Ras4B. Local structural analysis exhibits that GAP mainly reduces the conformational plasticity in the switch II of K-Ras4B relative to free K-Ras4B (Fig. 9B,C). This effect is also corroborated by analysis of the root-mean square fluctuations (RMSF) of K-Ras4B. As shown in Fig. 9D, GAP markedly mitigates the residue fluctuations in the switch II of K-Ras4B compared to the free K-Ras4B. We further determined the correlation of the displacements of all residue pairs of the free K-Ras4B (Fig. 9E) and the K-Ras4B–GAP complex (Fig. 9F) to examine motions of residues affected by GAP binding. The results show that, when compared to the free K-Ras4B, the domain-domain motions are significantly restricted in the K-Ras4B–GAP complex, particularly in the switch II domain. Collectively, these data suggest that in addition to providing the arginine finger R789 for catalysis, GAP has a profound influence on the conformational stability of the active, catalytically competent state of K-Ras4B.

### Oncogenic Mutations Disturb the Arrangements of Q61 and GAP’s R789, Impairing the GAP-mediated GTP Hydrolysis

**Figure 10:**
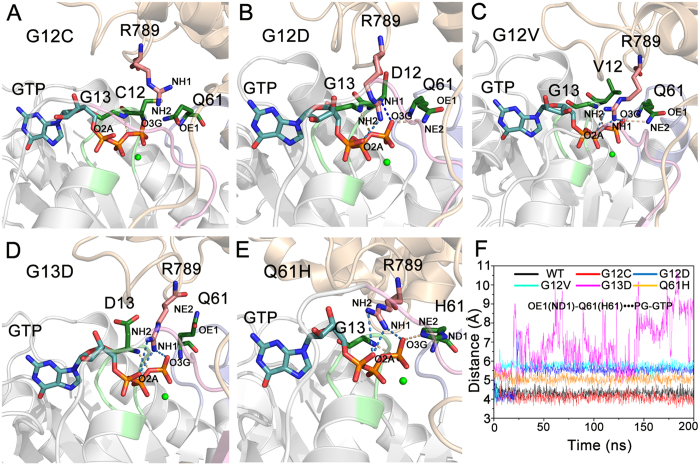
Oncogenic mutations disturb the catalytically-competent arrangements of Q61 and R789. Structural view of the active site from the representative structures of G12C (**A**), G12D (**B**), G12V (**C**), G13D (**D**) and Q61H (**E**) K-Ras4B-GTP**–**GAP complex. The salt bridge and H-bonding interactions are depicted by blue and wheat dotted lines, respectively. (**F**) Time dependence of the distance between Q61 OE_1_ atom and GTP P_γ_ atom (in Q61H mutant, the distance was measured between H61 ND_1_ atom and GTP P_γ_ atom) in the wild-type and oncogenic mutants. The G12C mutation significantly disturbs the arrangement of R789 where it moves away from GTP and of Q61, where it cannot form H-bond with the γ-phosphate. The G12D and G12V mutations cause rearrangement of the side chain OE_1_ atom of Q61 where it cannot extract a hydrogen atom from the catalytic water molecule. The G13D mutation abolishes the direct interactions between the side chain of Q61 and GTP. The Q61H mutation increases the distance between the side chain ND_1_ atom of H61 and γ-phosphorus and changes the angle among the atoms NE_2_ and ND_1_ of Q61 and γ-phosphorus, leading to the inability of Q61 to coordinate the catalytic water molecule.

The results above revealed that GAP not only provides the arginine finger R789, but also stabilizes the switch II with an accompanying positioning of catalytic residue Q61 in the active site for catalysis. To determine how oncogenic mutations impair the GAP-mediated GTP hydrolysis, the arrangements of R789 of GAP and Q61 of K-Ras4B in the oncogenic mutants were analyzed. Figure 10 shows the representative structure of K-Ras4B–GAP complex for each oncogenic mutant. Compared to the K-Ras4B^WT^-GTP-GAP complex (Fig. 8A), the G12C mutation markedly disturbs the arrangement of R789 where it moves away from GTP in the active site (Fig. 10A), while in other mutants the R789 remains engaged in salt bridge interactions with α- and γ-phosphates of GTP (Fig. 10B–E). This notion is supported by the distances between the side chain NH_2_ and NH_1_ atoms of R789 and the oxygen atoms of α- and γ-phosphates of GTP in the wild-type and oncogenic mutants (Fig. S4). As for the catalytic residue Q61, the side chain conformation of the residue is disturbed by oncogenic mutations. For example, the inability to form H-bond between the side chain NE_2_ atom of Q61 and the γ-phosphate of GTP is observed in the G12C mutant (Fig. 10A). The G12D and G12V mutations cause the side chain OE_1_ atom of Q61 where it extracts a hydrogen atom from the catalytic water molecule to move away from the γ-phosphorus of GTP (Fig. 10B,C). Notably, the G13D mutation disturbs the position of Q61, resulting in no direct interactions between the side chain of Q61 and GTP (Fig. 10D). Analysis of the distance between the side chain OE_1_ atom of Q61 and the γ-phosphorus of GTP also confirms these predictions (Fig. 10F). Furthermore, as shown in Fig. S5, analysis of the angle among the atoms NE_2_ and OE_1_ of Q61 and the γ-phosphorus of GTP indicates that the oncogenic G12 and G13 mutations disturb the side chain conformation of Q61, rendering it unable to interact with the catalytic water. However, in the oncogenic Q61H mutant, despite the existence of H-bonding interaction between the side chain NE_2_ atom of H61 and the γ-phosphate of GTP (Fig. 10E), the distance between the side chain ND_1_ atom of H61 and the γ-phosphorus of GTP increases (Fig. 10F) and the angle among the atoms NE_2_ and ND_1_ of Q61 and the γ-phosphorus of GTP changes as compared to the K-Ras4B^WT^-GTP–GAP complex (Fig. S5). These lead to the inability of the side chain of H61 to coordinate the catalytic water molecule. Taken together, these data indicate that the oncogenic mutations disturb the ‘correct’ arrangements of the catalytic residue Q61 of K-Ras4B and the arginine finger of R789 of GAP in the active site for catalysis, thereby impairing the GAP-mediated GTP hydrolysis

## Discussion

Ras proteins are proto-oncogenes that are frequently mutated in human cancers, such as lung, colon and pancreas^62^. Despite the more than three decades of efforts, no effective inhibitors of the Ras oncoproteins have been successful in the clinic, rendering the Ras proteins still ‘undruggable’^23^. The COSMIC database underscores the fact that aberrant Ras function in cancers is associated with a single mutation typically at residues G12, G13 and Q61^15^. Unraveling the mechanism through which oncogenic mutations affect the structural and dynamic behavior of Ras is expected to contribute to the development of targeted therapies for Ras-driven cancers.

A long-held view is that wild-type Ras is in the active state in its GTP-bound form that is capable of binding its effectors, including Raf, PI3K and RalGDS. Recent NMR and crystallographic data from Shima *et al*.^60^ have revised this notion. The authors found that GppNHp-bound H-Ras contains two interconverting conformations in solution, ‘inactive’ state and ‘active’ state, with the former bearing two inactive substates, 1 and 2. In a similar vein, our simulation of K-Ras4B^WT^-GTP suggests the existence of both active and inactive states (Figs S1 and 2A). Thus, these collective data argue that the conformational equilibrium between the two states may be common across members of the small GTPase family in their GTP-bound forms. The active state of K-Ras4B from our simulation resembles the ‘active’ state of H-Ras. However, only the lack of the interaction of G60 with the γ-phosphate, referred to as inactive substate 3, is observed in the inactive state of K-Ras4B, which is distinct from the two inactive substates of H-Ras described by both the uncoupling of T35 and G60 with the γ-phosphate or only the uncoupling of T35 with the γ-phosphate. In our timescales we did not capture the conformation of K-Ras4B^WT^-GTP visiting the two higher energy inactive substates, 1 and 2. The difference between the structures of the inactive substate 3 of K-Ras4B and the two inactive substates of H-Ras is attributed to that in H-Ras the T35 which is coordinated to Mg^2+^ was mutated to S35, which effectively captures large conformational changes in the switch I. Furthermore, the flexibility of switch II is much higher than switch I (Fig. 9B,C), revealing that the conformational transition of switch II is more accessible than that of switch I during a GDP/GTP exchange^41^,^63^. Based on the MD simulations, coupled with the crystallographic data, we suggest that the inactive state of K-Ras4B^WT^-GTP may exist in three substates in solution and the energy for the different states of K-Ras4B^WT^–GTP follows the order active state > inactive substate 3> inactive substate 2> inactive substate 1.

The effects of naturally occurring or experimentally generated point mutations in proteins on the redistributions of the conformational substates have been well-established^64^,^65^,^66^. The conformational analysis of the K-Ras4B–GTP in the wild-type and mutant states (Fig. 2) indicated that oncogenic mutations cause an inactive-to-active conformational transition. This results in higher population of active K-Ras4B–GTP in the oncogenic mutants than in the wild-type. However, the distinct oncogenic mutations trigger different dynamics, which may account for the different frequency and distribution of K-Ras4B mutations in human cancers. As shown in Fig. 2, the G12D, G12V, G13D, and Q61H mutations are more prone to shift K-Ras4B–GTP conformation to the active state than the G12C mutation. Moreover, as shown in Fig. 3, the G12C and G12D mutations cause larger conformational changes of K-Ras4B-GDP and result in higher exposure of the nucleotide-binding site as compared to the G12V, G13D and Q61H mutations. Taken together, the unique dynamics of the G12D mutant may explain why this oncogenic mutation is the most prevalent in K-Ras4B driven cancers^15^.

We further performed nucleotide exchange simulations of K-Ras4B^WT^ with the exchange of GTP to GDP to mimic the GTP → GDP hydrolysis. The loss of the γ-phosphate after GTP hydrolysis decouples the association of GDP and residues T35 and G60, favoring conformational changes of switch I and switch II to the open conformation which promotes the disassociation of GDP from its binding site. Our GTP-to-GDP exchange simulation was capable of partially capturing the conformational transition, with sampling of the GDP-like open conformation. However, the GDP-to-GTP exchange simulation was incapable of capturing the GTP-like closed conformation, revealing the difficulty encountered in visiting the higher energy state with conformational changes in the switch I and switch II regions. This difficulty may explain the requirement for proteins such as GEFs to execute the exchange of GDP with GTP^3^. GEFs accelerate the exchange reaction by several orders of magnitude. Overall, these simulations are consistent with K-Ras4B physiological processes. In resting cells, K-Ras4B is predominantly GDP-bound. Following growth factor stimulation^67^, GEF binding induces conformational changes in the two switch domains and the P-loop, catalyzing the exchange of GDP by GTP. Then, GAP binds to the active K-Ras4B–GTP to accelerate GTP hydrolysis, switching the protein to the inactive GDP-bound state to complete the catalytic process.

In solution, free K-Ras4B^WT^-GTP exists in active and inactive states. GAP binding promotes the transition of free K-Ras4B^WT^-GTP from the GTP-bound inactive to the GTP-bound active states (Figs 2A and 7), and stabilizes the intrinsically mobile K-Ras4B to correctly position the catalytic residue Q61, which in turn coordinates the catalytic water molecule (Figs 7 and 8). Because of the high flexibility of switch II in the GAP-free K-Ras4B, very little H-bonding interaction between Q61 and the GTP γ-phosphate is observed. However, in the K-Ras4B-GTP–GAP complex, the flexibility of switch II is significantly restricted by GAP (Fig. 9), which enables the formation of persistent H-bonding interaction between the Q61 and γ-phosphate of GTP (Fig. 8). However, the oncogenic mutations disturb the proper orientation of the GAP arginine finger R789 and the catalytic residue Q61 of K-Ras4B, leading to the mutated K-Ras4B resistance to GAP-mediated GTP hydrolysis. As a result, mutated K-Ras4B proteins persist in the active GTP-bound form that can interact with its downstream effector such as Raf leading to a sustained oncogenic signal^68^.

## Conclusion

Our MD simulations of wild-type and mutated K-Ras4B in the different states unraveled the mechanisms of how oncogenic mutations affect the GAP-mediated GTP hydrolysis as well as the mutational biases in K-Ras4B-driven cancers. Our results reveal that oncogenic mutations demolish the catalytically-competent conformations in the K-Ras4B–GAP complex, thereby impairing GAP-mediated GTP hydrolysis. When not bound to GAP, the mutations differentially elicit the K-Ras4B transition from the inactive to the active states in their GTP-bound forms and affect the dynamics of the nucleotide-binding site in their GDP-bound forms. These results provide insights into how oncogenic mutations affect the structural and dynamic behavior of K-Ras4B, help elucidate mutational biases in K-Ras4B-driven cancers and offer a potential venue for targeting K-Ras4B.

## Additional Information

**How to cite this article**: Lu, S. *et al*. The Structural Basis of Oncogenic Mutations G12, G13 and Q61 in Small GTPase K-Ras4B. *Sci. Rep*. **6**, 21949; doi: 10.1038/srep21949 (2016).

## Supplementary Material

Supplementary Information
