# Supplementary material for: The Structural Basis of Oncogenic Mutations G12, G13 and Q61 in Small GTPase K-Ras4B
Source: Sci Rep. 2016 Feb 23;6:21949. doi: 10.1038/srep21949 (PMC4763299; doi:10.1038/srep21949)
Supplement: Supplementary Information [file srep21949-s1.doc]

**Supporting Information**

**The Structural Basis of Oncogenic Mutations G12, G13 and Q61 in Small GTPase K-Ras4B**

Shaoyong Lu^1,2^ Hyunbum Jang^2^, Ruth Nussinov^2,3^,*, and Jian Zhang^1^,*

^1^Department of Pathophysiology, Key Laboratory of Cell Differentiation and Apoptosis of Chinese Ministry of Education, Shanghai Jiao Tong University, School of Medicine, Shanghai, 200025, China

^2^Cancer and Inflammation Program, Leidos Biomedical Research, Inc., Frederick National Laboratory, National Cancer Institute, Frederick, MD 21702, USA

^3^Department of Human Genetics and Molecular Medicine, Sackler School of Medicine, Sackler Institute of Molecular Medicine, Tel Aviv University, Tel Aviv 69978, Israel

**Corresponding Author:** *J.Z.: e-mail, [jian.zhang@sjtu.edu.cn](mailto:jian.zhang@sjtu.edu.cn); *R.N.: e-mail, [NussinoR@helix.nih.gov](mailto:NussinoR@helix.nih.gov); Telephone: 301-846-5579; Fax: 301-846-5598.

**Table S1** Summary of MD simulation systems

| *System name* | *Ions* | *Water molecules* | *Total*  *atoms* | *Simulation time (ns)* |
| --- | --- | --- | --- | --- |
| **Simulation of monomer K-Ras4B** | | | | |
| Wild-type K-Ras4B-GTP | 9 | 5,003 | 17,689 | 400 |
| G12C K-Ras4B-GTP | 9 | 4,962 | 17,570 | 400 |
| G12D K-Ras4B-GTP | 10 | 4,959 | 17,563 | 400 |
| G12V K-Ras4B-GTP | 9 | 4,954 | 17,551 | 400 |
| G13D K-Ras4B-GTP | 10 | 5,000 | 17,686 | 400 |
| Q61H K-Ras4B-GTP | 9 | 5,013 | 17,719 | 400 |
| Wild-type K-Ras4B-GDP | 8 | 6,555 | 22,340 | 400 |
| G12C K-Ras4B-GDP | 8 | 5,749 | 19,926 | 400 |
| G12D K-Ras4B-GDP | 9 | 5,746 | 19,919 | 400 |
| G12V K-Ras4B-GDP | 8 | 5,748 | 19,928 | 400 |
| G13D K-Ras4B-GDP | 9 | 6,551 | 22,334 | 400 |
| Q61H K-Ras4B-GDP | 8 | 6,557 | 22,346 | 400 |
| **Nucleotide exchange simulation** | | | | |
| Wild-type K-Ras4B with the exchange of GTP by GDP | 8 | 5,367 | 18,776 | 200 |
| Wild-type K-Ras4B with the exchange of GDP by GTP | 9 | 5,556 | 19,348 | 200 |
| **Simulation of K-Ras4B-GTP–GAP** | | | | |
| Wild-type K-Ras4B-GTP–GAP | 15 | 17,262 | 59,647 | 200 |
| G12C K-Ras4B-GTP–GAP | 15 | 17,263 | 59,654 | 200 |
| G12D K-Ras4B-GTP–GAP | 16 | 17,262 | 59,653 | 200 |
| G12V K-Ras4B-GTP–GAP | 15 | 17,263 | 59,659 | 200 |
| G13D K-Ras4B-GTP–GAP | 16 | 17,262 | 59,653 | 200 |
| Q61H K-Ras4B-GTP–GAP | 15 | 17,262 | 59,647 | 200 |
| Totally 6400 | | | | |


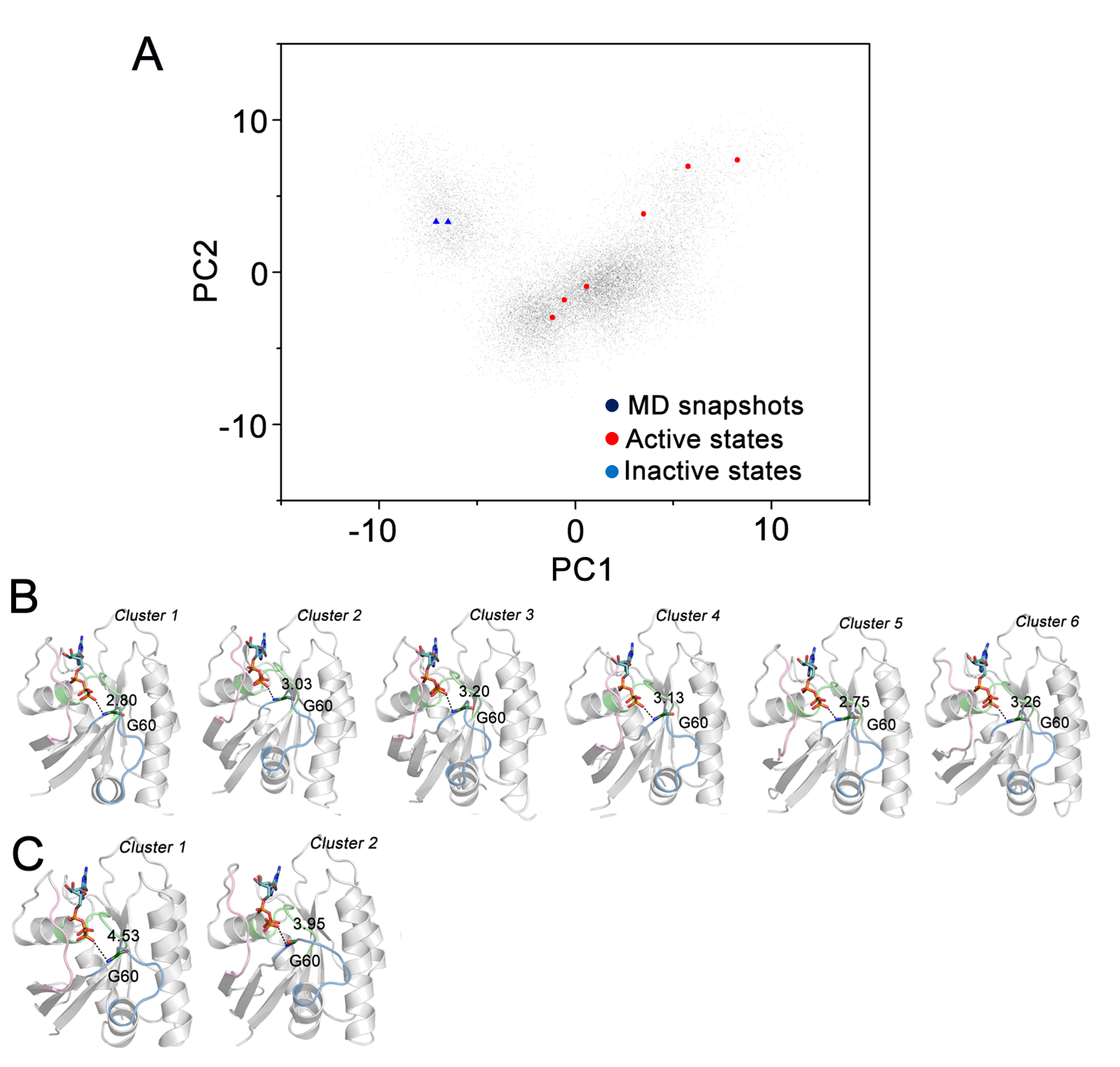


**Figure S1:** (A) Results of principle component analysis (PCA) on the K-Ras4B^WT^-GTP. MD snapshots are projected onto the first and second principle components (PC). The representative structures obtained from MD trajectory using cluster analysis are projected onto the first two PCs. The green circles represent active states, while the blue circles represent inactive states. (B) The detailed representative structures from the six active clusters. (C) The detailed representative structures from the two inactive clusters. The distance (Ǻ) between the oxygen atom of γ-phosphate and the G60 N atom is labeled for each representative structure. The distance is much larger in the inactive states than in the active states, revealing the loss of G60-γ-phosphate interaction in the inactive states.


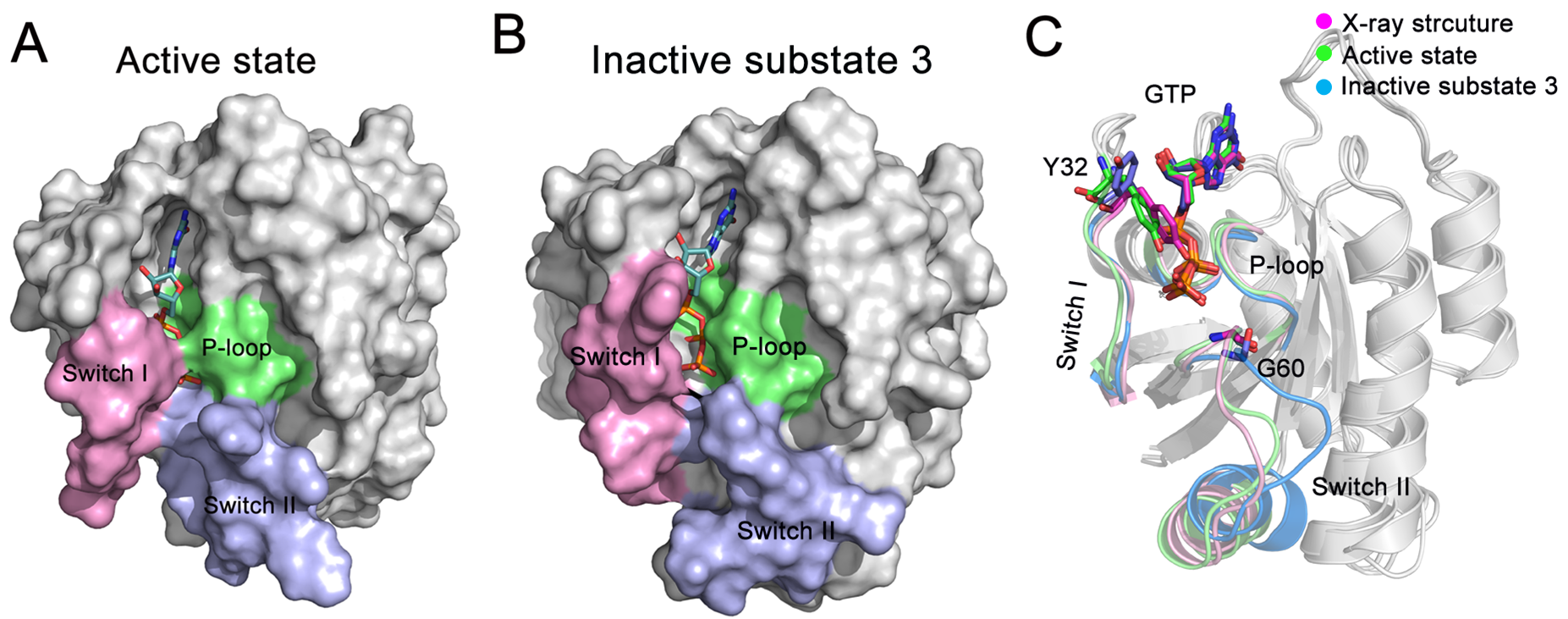


**Figure S2:** Surface representation of the representative structures of the active state (A) and the inactive state 3 (B) of K-Ras4B^WT^-GTP. (C) Backbone superimposition of the active state (green) and the inactive substate 3 (light blue) to the crystal structure (magenta) of GppNHp-bound K-Ras4B.


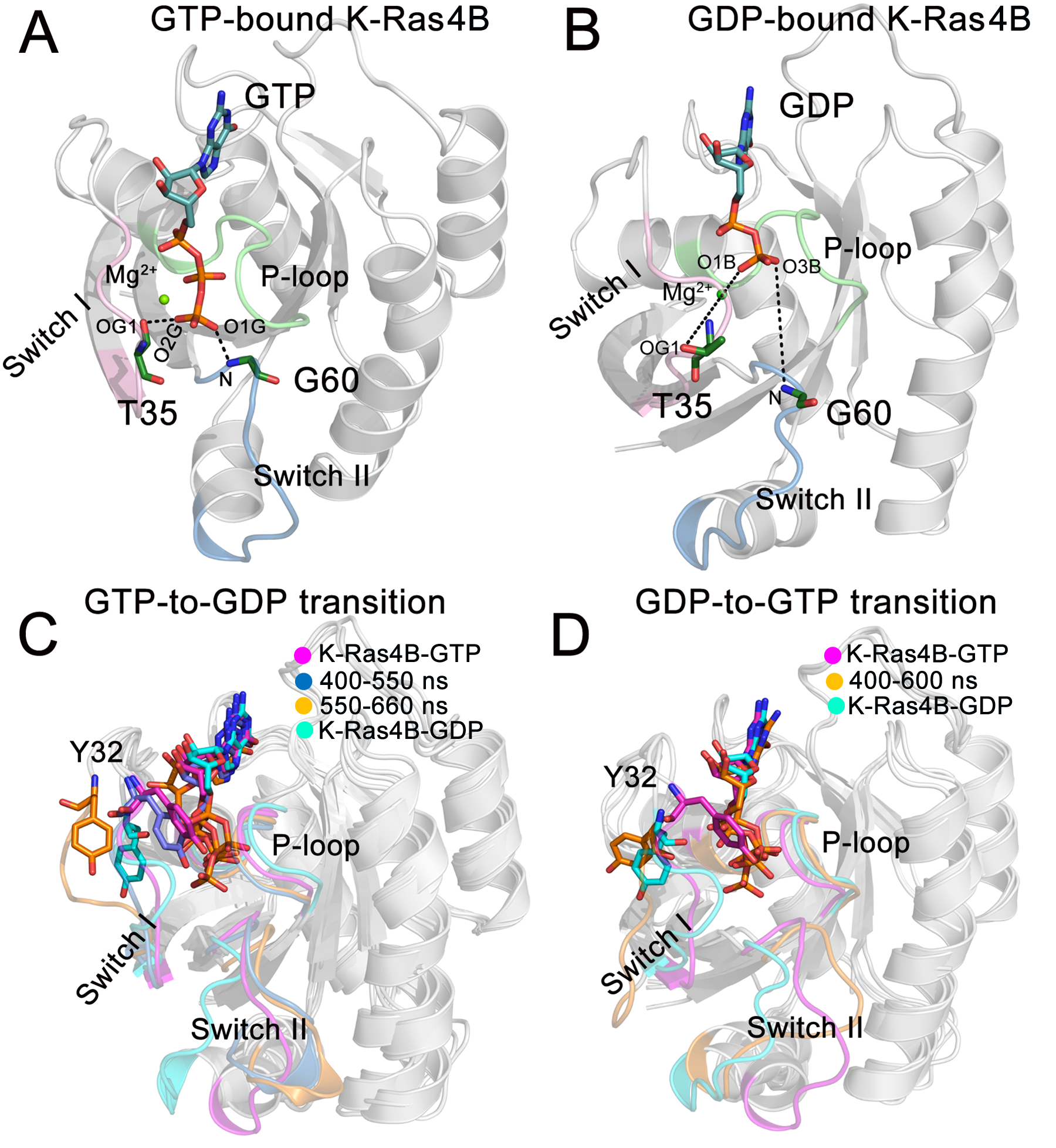


**Figure S3:** (A) The direct interactions of T35 and G60 with the γ-phosphate of GTP in the GTP-bound K-Ras4B. (B) No direct interactions of T35 and G60 with the β-phosphate of GDP in the GDP-bound K-Ras4B. (C) Backbone superimposition of the two representative structures derived from 400-550 ns (blue) and 550-600 ns (orange) in the GTP-to-GDP exchange simulation to the crystal structures of GppNHp- (magenta) and GDP-bound (cyan) K-Ras4B. (D) Backbone superimposition of the representative structure derived from 400-600 ns (orange) in the GDP-to-GTP exchange simulation to the crystal structures of GppNHp- (magenta) and GDP-bound (cyan) K-Ras4B.


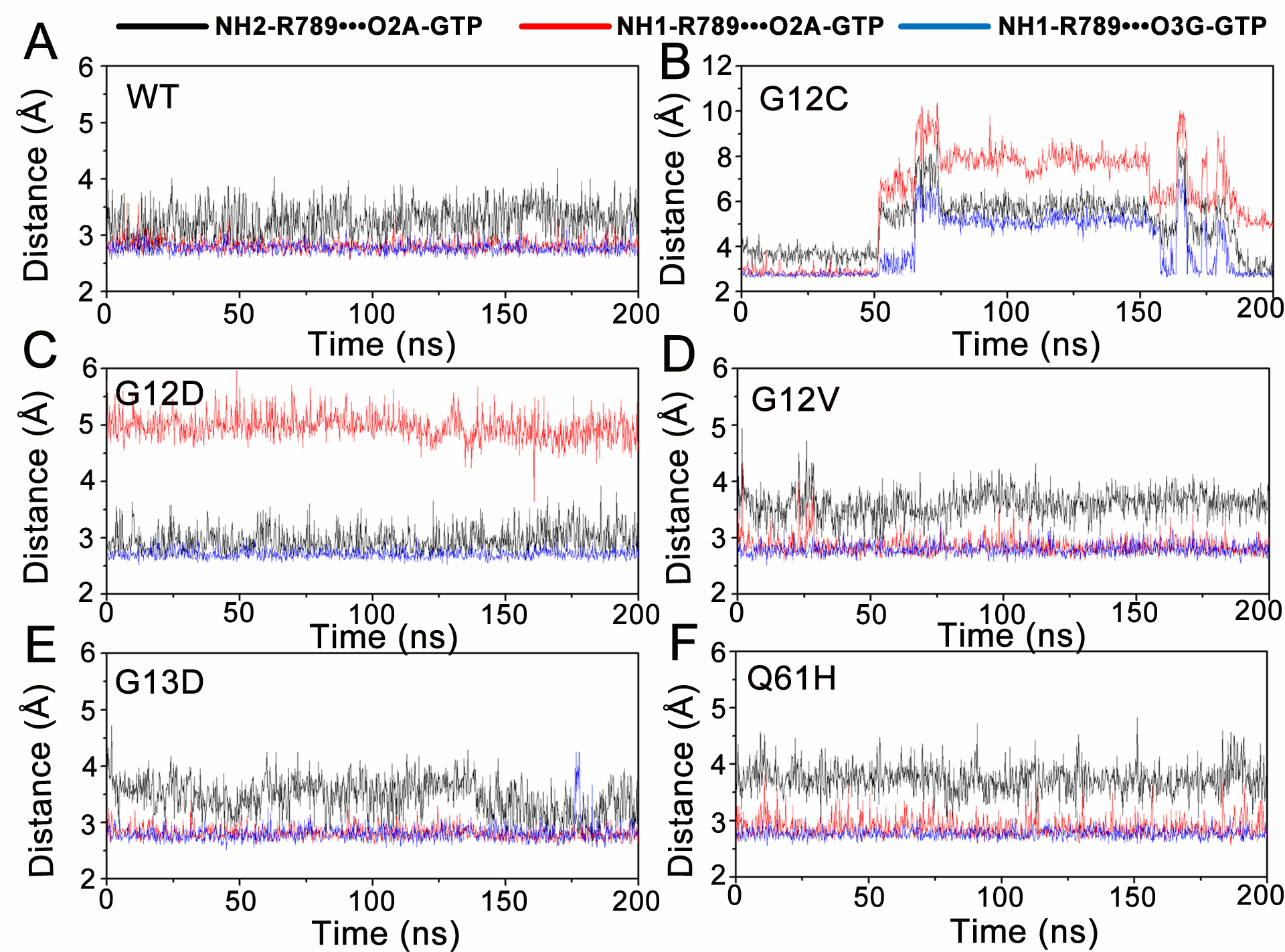


**Figure S4:** Time dependence of distances of R789 NH2 atom to GTP O2A atom (black), of R789 NH1 atom to GTP O2A atom (red), and of R789 NH1 atom to GTP O3G atom (blue) in the wild-type (A), G12C (B), G12D (C), G12V (D), G13D (E) and Q61H (F) K-Ras4B-GTP–GAP complex.


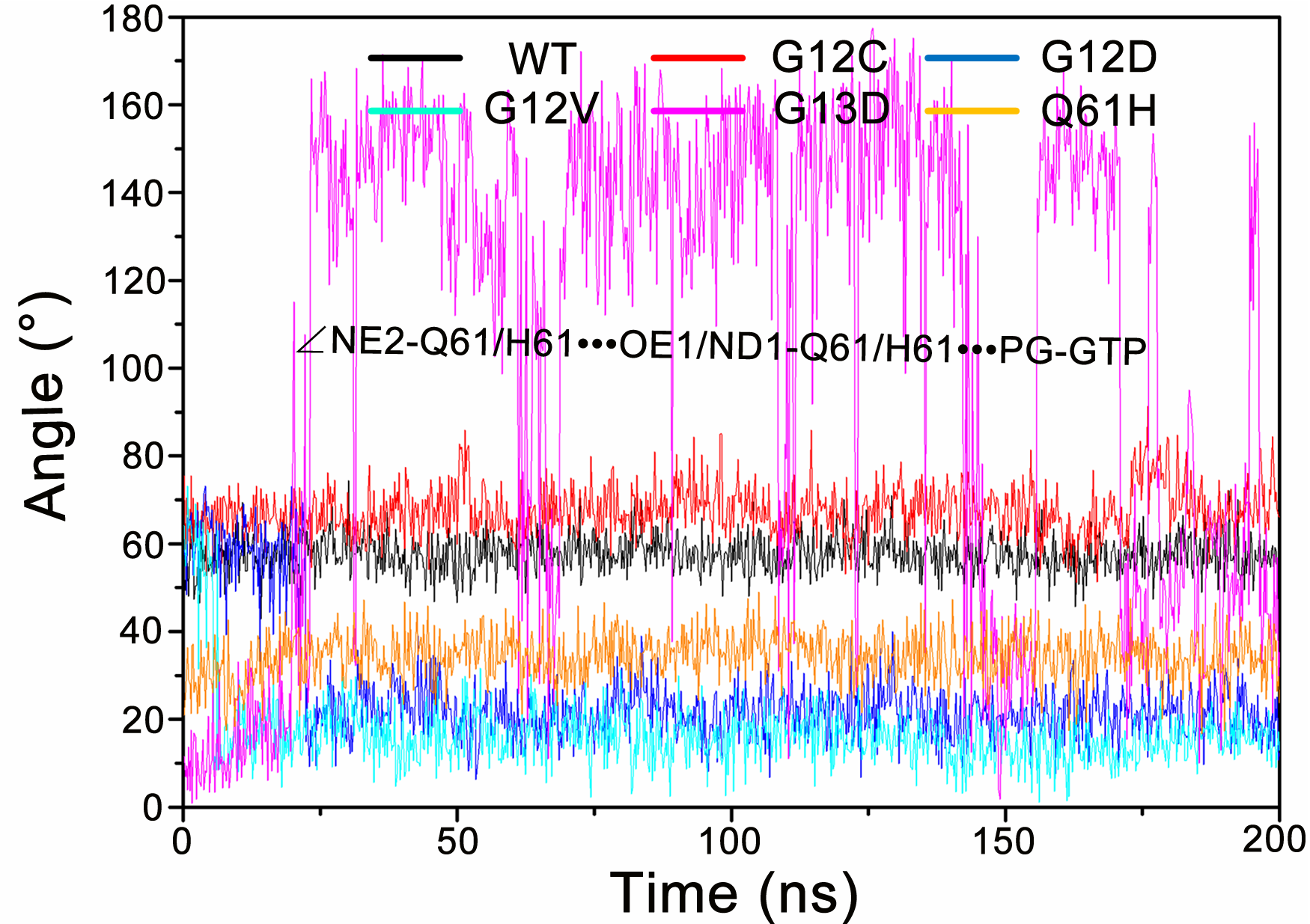


**Figure S5:** The angle among the atoms NE_2_ and OE_1_ of Q61 and GTP P_γ_ atom (in Q61H mutant, the angle was measured among the atoms NE_2_ and ND_1_ of Q61 and GTP P_γ_ atom) in the wild-type and oncogenic mutants of K-Ras4B-GTP–GAP.
